# Curriculum Research: Disseminating Neuropalliative Care Education Through an Adaptable Curriculum: A Multisite Feasibility Trial

**DOI:** 10.1212/NE9.0000000000200133

**Published:** 2024-05-21

**Authors:** Eileen Harrigan, Breana L. Taylor, Hannah L. Kirsch, Shivani Ghoshal, Kimberly T. Kwei, Kate T. Brizzi, Claire J. Creutzfeldt, Tarini Goyal

**Affiliations:** From the Division of Geriatric Medicine and Palliative Care (E.H.), Department of Medicine, NYU Langone Health, New York; Department of Neurology (B.L.T., C.J.C.), University of Washington, Seattle; Department of Neurology & Neurological Sciences (H.L.K.), Stanford University School of Medicine, Palo Alto, CA; Department of Neurology (S.G., K.T.K., T.G.), New York Presbyterian-Columbia University Irving Medical Center, NY; Division of Palliative Care (K.T.B.), Departments of Neurology and Medicine, Massachusetts General Hospital, Boston; and Cambia Palliative Care Center of Excellence (C.J.C.), University of Washington, Seattle.

## Abstract

**Introduction and Problem Statement:**

Neurologic disease is a leading cause of disability and death worldwide. As the global population ages, the burden of these diseases is expected to increase. Despite this increased clinical need, neurology trainees are seldom taught skills and concepts in palliative care. Education in Palliative and End-of-Life Care for Neurology (EPEC-N) is a publicly available neuropalliative care curriculum designed to be taught by both palliative care specialists and nonspecialists alike.

**Objectives:**

(1) To create a feasible curriculum in neuropalliative care using EPEC-N, (2) to improve learners' satisfaction with neuropalliative care training, and (3) to improve learners' confidence with neuropalliative care topics.

**Methods and Curriculum Description:**

Three academic centers implemented a neuropalliative care curriculum for neurology residents using EPEC-N modules. Each site selected 4 of the 26 topics. Instructor backgrounds varied by site and included neurology senior residents, fellows, and faculty; none had completed palliative care fellowship at the time of teaching. Teaching methods included lecture, case discussion, and role-play. To assess feasibility and acceptability of this curriculum, learners, instructors, and site leads completed postsession surveys.

**Results and Assessment Data:**

A total of 87 residents attended at least 1 didactic session, and 23 residents completed the evaluation survey. All 3 sites were able to successfully implement an evidence-based and subspecialist-approved neuropalliative care curriculum without relying on subspecialty instruction, despite variations in instructor background, curriculum format, and module selection. Learners overall expressed a positive experience with this curriculum, with most of the respondents indicating that each session was effective in improving their knowledge base, relevant to current practice, and provided in an effective teaching format. Site leads and instructors found the curriculum easy to use, in minimal need of modification, and helpful for delivering neuropalliative care education.

**Discussion and Lessons Learned:**

The EPEC-N curriculum was successfully implemented at 3 US sites, demonstrating feasibility, acceptability, and adaptability across institutions. Further research is needed to evaluate the effectiveness of this curriculum in improving neuropalliative care skills for neurologists and raising the standard of primary neuropalliative care.

## Introduction and Problem Statement

Neurologic illness is the leading cause of disability and the second leading cause of death globally. The number of disability-adjusted life years due to neurologic illness exceeds 270 million, while approximately 9 million deaths were attributable to neurologic disease in 2016.^1^ Patients with these diseases, including dementia, movement disorders, neuromuscular disease, stroke, and brain tumors, have a wide array of unmet palliative care needs, ranging from symptom management to caregiver burden and end-of-life care. As the global population ages, the number of patients with neurologic disease is expected to increase. This will result in a growing need for care that manages symptoms and functional impairment as well as the psychosocial and spiritual needs of patients and families. For most patients with primary neurologic diagnoses, addressing these concerns falls to the patient's neurologist, comprising a care model known as primary palliative care.^2^

In 2022, the American Academy of Neurology published a position statement on neuropalliative care needs and goals which identified gaps in current neuropalliative care education, including a need to “identify the best educational approaches to empower neurologists to practice effective primary palliative care.”^3^ In 2019, the American Council for Graduate Medical Education formally recommended specific training in palliative care for neurology residents for the first time.^4^ This updated recommendation reflects the growing recognition of neurologists' key role in primary palliative care, including serious illness communication.^5,6^ Resident education has been repeatedly highlighted in the literature as a priority in the field of neuropalliative care,^7,8^ and a survey of neurology residency program directors demonstrated that only 42% were satisfied with palliative care education in their program.^10^ Time for teaching, availability of faculty, and faculty expertise were cited as the most common barriers.^10^ There are relatively few neuropalliative care specialists available, highlighting the need for neurologists without formal training to teach trainees.^7^

In a recent study^9^ VitalTalk^20^ (a well-known and effective program for teaching serious illness communication skills) was successfully adapted and implemented for neurology residents with the aim of improving residents' communication skills and confidence. This study used a multimodal curriculum with didactics and simulated patient encounters, and was taught by internal medicine and neurology faculty with palliative care postgraduate training. However, the availability of neuropalliative care experts and access to standardized patients may limit other programs' ability to replicate such a curriculum. There is a need for accessible, adaptable neuropalliative care curricula that can be taught by neurologists with or without formal palliative care training.

## Objectives

In this study, we use the Education in Palliative and End-of-Life Care for Neurology (EPEC-N) program as an adaptable curriculum to teach neurology residents at multiple institutions with different educational resources. The EPEC-N curriculum is a potential resource for neurology programs around the world to train residents and other learners in primary palliative care, with or without access to neuropalliative care specialists. By having both disease-specific and communication-specific modules, this resource can be tailored to suit the needs of learners from various fields, clinical contexts, and training backgrounds. In this study, we evaluate the feasibility and acceptability of adapting and implementing the EPEC-N curriculum to teach neuropalliative care at 3 neurology residency programs in the United States, each with different instructor backgrounds and programmatic resources. Our objectives were to

1. create a feasible curriculum implementation experience for instructors,
2. improve learners' satisfaction with their neuropalliative care training, and
3. improve learners' confidence with neuropalliative care topics.

## Methods and Curriculum Description

### Standard Protocol Approvals, Registrations, and Consents

The study was reviewed by the Institutional Review Boards at Columbia University, University of Washington, and Stanford University and determined to be exempt based on the standard exemption protocol for research conducted in established educational settings involving normal educational practices.

### Curriculum Background and Content

The EPEC program was developed more than 20 years ago and has demonstrated the efficacy of a train-the-trainer model in national dissemination of general palliative care education and has successfully produced adapted curricula for multiple specialties, including oncology, emergency medicine, and pediatrics.^11-13^ Based on a neuropalliative care textbook,^14^ the International Neuropalliative Care Society has developed a neuropalliative care curriculum, EPEC-N,^15^ that the society has made available to the public at no cost (the society is supported by membership fees and donations). The curriculum consists of 26 modules (Table 1), each addressing a specific neuropalliative care topic, written by experts in the field and available for download in English or Spanish. Each module consists of an annotated slide set, including notes for the presenter and suggested teaching techniques, and a list of key references. A preliminary study with community neurologists demonstrated improvement in their comfort with palliative care after taking a course comprised of EPEC-N modules.^16^

**Table 1:** Complete List of Available EPEC-N Modules Through International Neuropalliative Care Society^13^

Category	Available modules
General overview	Introduction to neuropalliative care
	Pediatric neuropalliative care
	Neuropalliative care career paths and care models
	Teaching with EPEC-N
	Hospice care in neurologic disease
	Caregiver assessment and support
Disease-specific	Severe acute brain injury
	Disorders of consciousness
	Parkinson disease and related disorders
	Dementia
	Multiple sclerosis
	Neuromuscular diseases
	Neuro-oncology
	Epilepsy
Communication	Improving medical decisions
	Communication and delivering difficult news
	Prognostication in neurologic disease
	Working with families
	Goals of care
Ethics	Advance care planning
	Withdrawing or withholding life sustaining therapies
	Responding to requests for hastened death
Symptoms	Pain assessment and management
	Cannabinoids for neurologic disorders
Spirituality and self-care	Spirituality in palliative care
	Self-care for clinicians

Abbreviation: EPEC-N = Education in Palliative and End-of-Life Care for Neurology.

### Curriculum Structure

Each site (from here referred to as site 1, site 2, and site 3, respectively) had a designated “site lead” who recruited instructors, guided the selection of at least 4 of 26 available modules from EPEC-N, and distributed surveys to learners and instructors (eAppendices 1 and 2). Site leads were asked to implement 4 modules because preliminary interviews with residency leadership showed that incorporating 4 sessions into existing resident didactics would be viable without infringing on clinical time and would permit a combination of communication-focused and disease-specific topics as desired. Methods for selecting modules varied by site. At site 1, a needs assessment survey was conducted, wherein learners were given a list of all 26 modules and selected the topics that they would like to learn about based on their self-perceived needs. The top 4 modules selected were then implemented in the curriculum. Site 2 selected modules based on the clinical and academic interest of instructors recruited and commonly seen pathology on clinical teaching services; no structured needs-assessment survey was applied. Disease-specific modules were chosen because there was a preexisting communication skills workshop for residents. At site 3, 8 modules were selected based on perceived relevance to the residents' current curriculum and residents were asked to select the 4 topics to fill self-perceived need. No specific needs assessment framework was otherwise used. All 3 sites are tertiary care academic medical centers. Sites 1 and 2 are in an urban setting, and site 3 is suburban.

Attendance was not recorded for each session, so the exact capture rate for each topic is unknown. Instructor background varied by site and included a mix of neurology senior residents, fellows, and faculty who were selected based on academic and clinical interest in relevant neuropalliative care topics. Several instructors participated in the Becoming and EPEC Trainer course, wherein participants learn various strategies for teaching and implementing palliative care education; a trainer course specific to neuropalliative care has since been developed.^17^ Each instructor was given an EPEC-N slide set and had the opportunity to modify the module to their preferred content and teaching style. A visual representation of module selection, instructor background, and curriculum structure at each site is available in eTable 1 and is detailed below.

#### Site 1

All 30 adult neurology residents were invited to participate in the study. The curriculum was delivered using 1-hour virtual small group sessions over the course of the 2021–2022 academic year; small group sessions with 3–6 residents are incorporated into the regular resident didactic schedule. Each module was repeated 6 times to capture all available residents. Teaching methods included lecture, case-based discussion, and role-play. Instructors were either neurology faculty (1 neurohospitalist, 1 neurointensivist, 1 neuro-oncologist, and 1 movement disorders specialist) or a neurology trainee (1 neurology postgraduate year [PGY]-4 resident planning to pursue hospice and palliative medicine fellowship training). We conducted a needs assessment, performed through emailed survey, of residents to select the modules. Selected modules were (1) communication and delivering difficult news, (2) neuro-oncology, (3) advance care planning, and (4) goals of care.

#### Site 2

All adult neurology residents and junior pediatric neurology residents were invited to participate in the study, a total of 27. The neurology residency program has weekly 2-hour didactics each for junior (PGY2) and for senior (PGY3/4) neurology residents. Four modules were presented during the usual resident didactics using lecture and case-based discussion. Some sessions were in-person, while others were given virtually. Each module was given twice, once during junior didactics and once during senior didactics. The instructors were 2 senior neurology residents, a vascular neurology fellow and a neurocritical care fellow, each of whom selected a module they felt most comfortable with. Selected modules were (1) severe acute brain injury (SABI), (2) multiple sclerosis, (3) Parkinson disease and related disorders, and (4) disorders of consciousness.

#### Site 3

All adult neurology residents were invited to participate in the study, a total of 30. The curriculum was delivered in 1-hour sessions as part of regularly scheduled resident didactics, in a hybrid in-person/virtual format. Sessions were recorded and could also be viewed afterward. All sessions were taught by a neurointensivist with an interest in neuropalliative care and education, using lecture and case-based discussion. Modules were selected by residents through a precurriculum needs assessment survey and included (1) prognostication in neurologic disease, (2) withdrawing or withholding life-sustaining therapies, (3) pain assessment and management, and (4) SABI.

### Curriculum Assessment

Our primary outcome was feasibility of implementing a neuropalliative care curriculum. Secondary outcomes included learner satisfaction and self-rated knowledge. Postcurriculum survey assessments were developed to assess the curriculum's feasibility and acceptability for learners and instructors (eTable 2). Surveys were designed and distributed through Qualtrics (Provo, UT) and consisted of multiple-choice basic demographic questions, Likert-type assessments of each module, and open-ended questions eliciting perceived strengths and shortcomings of the material. Likert-type questions were rated as “strongly agree,” “agree,” “disagree,” “strongly disagree,” and “did not attend/do not recall.” Answers were then dichotomized into categories of “agree” (if “strongly agree” or “agree” were selected) or “disagree” (if “disagree” or “strongly disagree” were selected). Those who did not recall or attend the session were excluded from the analysis for that session. Surveys were distributed to all learners and instructors through email following completion of all 4 modules of the curriculum and consisted of the same questions at all 4 sites. Survey questions were adapted from the original EPEC materials^11^ and did not use a specific framework. The learner and instructor survey assessment questionnaires are included in supplemental materials.

Given the design of this study, each program's curriculum had different learning objectives depending on which modules were used (eTable 3). Examples from 1 disease-focused and 1 communication-focused module are given in Table 2.

**Table 2:** Learning Objectives From 2 Modules in EPEC-N

SABI	Communication and delivering difficult news
1. Define SABI 2. Describe the care trajectory of patients with SABI 3. Identify symptom management needs for patients and support needs for families	1. Reflect on the effect of skilled and compassionate communication on patient care and provider satisfaction 2. Describe skills to address barriers to serious illness communication 3. Identify the triggers for serious illness communication 4. Implement the SPIKES framework for delivering “difficult news” 5. Use NURSE statements to respond to emotion 6. Engage in role-play to practice communication tasks

Abbreviations: EPEC-N = Education in Palliative and End-of-Life Care for Neurology; SABI = severe acute brain injury.

### Data Availability

Surveys and results not published within this article will be shared with any interested investigator by request.

## Results and Assessment Data

### Feasibility of Curriculum Implementation

Formal attendance was not taken for each session. Participation in the postintervention survey is summarized in Table 3.

**Table 3:** Learner Demographics and Survey Participation

No. of learners responding to survey	Site 1 (n = 8)	Site 2 (n = 10)	Site 3 (n = 5)	Total (n = 23)
PGY-2	n = 1	n = 9	n = 1	n = 11
PGY-3	n = 4	n = 0	n = 2	n = 6
PGY-4	n = 3	n = 1	n = 2	n = 6

Abbreviation: PGY = postgraduate year.

Site 1 offered this curriculum to 30 residents, while site 2 offered this to 27 residents, and site 3 to 30 residents.

Overall, site leads reported a positive experience using EPEC-N to implement a neuropalliative care curriculum and all of them plan to use this resource again. In open-ended feedback, site leads repeatedly cited the “ease of use” of the modules, stating “Having the talks pre-created made it easy to recruit instructors. The variety of topics also helped expand what we could offer here that is outside of our subspecialty expertise.” They also saw the curriculum's structure as helpful, stating that this curriculum “provided an organizational framework for instructors, especially when it comes to designing a sustainable set of lectures.” According to site leads, a logistical advantage was the ability to integrate the curriculum into the existing resident learning schedules. Despite this, site leads noted that not all residents attended each session because of clinical schedules, illness, or vacation.

Five of the 10 total instructors responded to surveys. Preparation for sessions took less than 3 hours for all instructors, and a minority (40%) of instructors made modifications to the slides, including adding opportunities for role-play and reducing redundancy. All instructors reported that EPEC-N facilitated their teaching and contributed to their comfort teaching neuropalliative care. In open-ended feedback (Table 4), instructors appreciated having a framework and evidence-based slide decks for teaching outside of their subspecialty, and believed more case-based discussion would be useful, with 1 instructor stating, “I'd have more built in case examples and audience participation opportunities.”

**Table 4:** Summary of Common Comments From Open-Ended Survey Questions

	What was effective about the neuropalliative care modules?	What would have made the neuropalliative care modules more effective?
Learners	• Relevant content • Helpful communication approach, particularly for SABI	• More interactive teaching methods (case-based discussion, greater practice with role-play, simulation) • Content suggestions • Include other topics such as cultural differences and medicolegal aspects of palliative care • Exclude less relevant topics such as outpatient opiate management • Should be given earlier in residency
Instructors	• Well-organized • Easy to modify • Has broad range of neuropalliative care content	• Content too broad, not necessarily applicable to neurology • Should include more practical skills • More learner participation • Improved graphics
Site leads	• Convenient to have premade slides • Variety of topics, not limited by local subspeciality availability • Well organized	• More concrete communication skills • More case-based discussion • Design of slides should be improved

Abbreviation: SABI = severe acute brain injury.

### Learner Satisfaction and Self-Rated Knowledge

**Figure 1:**
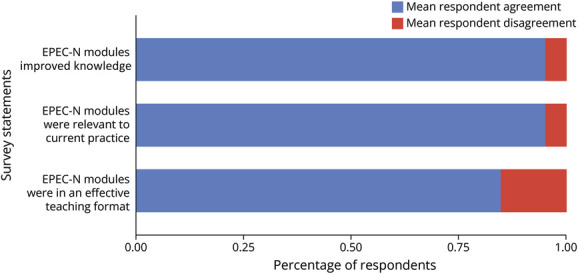
Overall Learner Satisfaction Learner satisfaction rates with EPEC-N modules according to the percentage of residents who agree with survey statements, averaged across all modules taught. EPEC-N = Education in Palliative and End-of-Life Care for Neurology.

**Figure 2:**
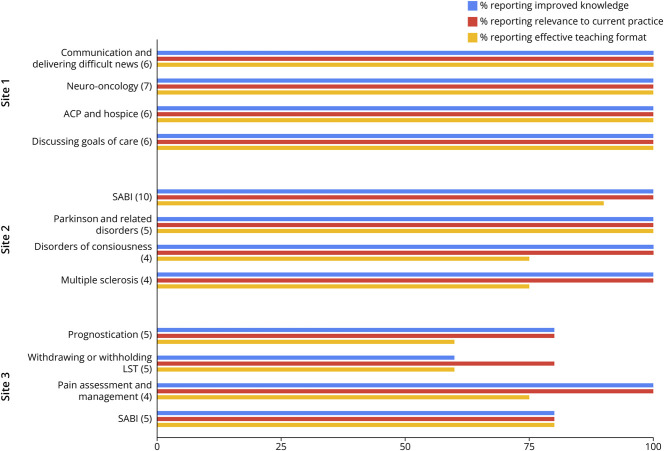
Learner Responses Learner survey responses for each didactic session, grouped according to site of instruction. The first panel corresponds to sessions conducted at site 1, followed by site 2 results, and finally site 3 results. Data in parentheses represent the “n” for each educational session. ACP = advance care planning; LST = life-sustaining treatment; SABI = severe acute brain injury.

Overall, learners expressed a positive experience with this curriculum, with the majority of respondents indicating that each session was effective in improving their knowledge base (mean 93.3%), were relevant to current practice (mean 95%), and were in an effective teaching format (mean 84.6%) (Figure 1). Figure 2 demonstrates learner satisfaction of each individual module, according to improvement in knowledge, relevance to current practice, and efficacy of teaching format. The only module that was repeated across more than 1 site was SABI, which was delivered at sites 2 and 3 and was more favorably received by learners at site 2 than at site 3 in all survey categories (Figure 2). Table 4 summarizes open-ended responses from learners. Respondents expressed appreciation for learning communication techniques and felt they had improved understanding of their role as neurologists in serious illness conversations.

When asked what could be improved, residents requested starting sessions earlier in their training, given that they often have early involvement in serious illness conversations. Residents were appreciative of and interested in interactive learning styles, requesting strategies such as “flipped classroom” and case-based discussion. Some residents expressed a desire for more teaching on end-of-life symptom management, the legal aspects of end-of-life care and planning, and cultural practices and beliefs about the dying process.

## Discussion and Lessons Learned

The EPEC-N curriculum was successfully implemented at 3 different neurology residency programs using the existing resident learning schedules, demonstrating the potential for using EPEC-N as a starting point for establishing neuropalliative care curricula in neurology residency programs nationwide. Respondents overall found this curriculum acceptable, both as learners and instructors, and learners reported improved knowledge in neuropalliative care topics. All 3 sites created distinct, customized curricula using EPEC-N, highlighting its adaptability.

The sites were different in module selection, instructor background and previous experience, and teaching schedule. Learners felt that the modules improved their knowledge and were relevant to their practice regardless of whether the topics were more communication-focused (site 1), disease-specific (site 2), or a combination (site 3). In instructor background, none of the instructors had completed fellowship in palliative medicine, and a minority of instructors had previous experience teaching some type of palliative care content. Instructors at sites 1 and 3 had completed the “Becoming an EPEC trainer” teaching workshop. Finally, teaching format and teaching techniques varied across sites. All 3 sites used lecture and case-based discussion as the core teaching techniques, although site 1 also incorporated role-play. Group size for learners varied, with site 1 having the smallest groups (4–5 residents per session), site 2 with larger groups (junior residents in one and senior residents in another), and site 3 with the largest (all residents together). Learners at site 1 more consistently found the teaching format to be effective compared with other sites. This may be related to the small group size, which could facilitate greater discussion and peer learning, or to the incorporation of role-play techniques. A majority of learners at site 3 found the curriculum acceptable and effective but had a lower response rate and overall rated the modules less highly than at other sites. Of note, residents have a preexisting communication skill curriculum and receive individual communication coaching at site 3. This, along with larger instruction groups for this experimental curriculum, may have affected their rating of effectiveness of additional palliative care training and their self-rated knowledge enhancement. Of note, however, module topics were selected with their input.

This study is unique in examining the instructor experience, a key component to a feasible, sustainable curriculum. All instructors found the EPEC-N materials helpful and spent less than 3 hours preparing to teach. Site leads, who recruited instructors and organized the curriculum, reported that the existence of the EPEC-N modules made it easier to recruit instructors because they did not have to prepare original materials. The most compelling evidence of the curriculum's utility is that all 3 sites plan to use EPEC-N again. Neurology program directors have previously cited time for teaching, faculty availability, and faculty expertise as barriers to implementing neuropalliative care teaching^10^; these barriers can all potentially be addressed by incorporation of EPEC-N into existing teaching schedules.

There are several limitations to our study. First and most significant is the low response rate of learner and instructor survey, consistent with the known phenomenon of low response rates for surveys of health care providers.^17^ Possible explanations for this include residents' typically busy schedule and “survey fatigue.” This limits the generalizability of our data, although the overall positive feedback from all 3 sites may be an indicator toward generalizability. We are unable to exclude the possibility of a nonresponse bias because systematically comparing the respondents with nonrespondents was beyond the scope of this work. For future studies, we would plan to use strategies for optimizing response rates, such as incentives, sharing common goals between surveyors and respondents, tracking initial and follow-up communication, and using persistent but contextual follow-up methods.^18^ Response rates for the surveys varied by module and ranged from 5 to 10 responding learners. No discernible pattern could be identified regarding module topic or methodology and this variability in response rate; therefore, this variation may reflect differences in attendance rates at specific sessions. Second, we did not track attendance at the sessions and so do not know the exact number of residents who were able to attend all portions of the curriculum. However, an advantage of all 3 sites integrating the curriculum into the usual resident learning schedule is that attendance was likely optimized, compared with a stand-alone workshop without protected nonclinical time. Third, our study primarily aimed to address feasibility and acceptability, with subjective immediate learning as a secondary outcome. These data correspond to the first and second levels of Kirkpatrick training assessments—reaction and learning, which are foundational to learning but do not represent a rigorous method of clinical skill evaluation.^20^ We did not address objective measures of learning such as formal skills or knowledge assessments, which are better assessed with techniques such as standardized patient encounters and simulation sessions. Further studies will be needed to assess higher levels of efficacy, including behavior change observed by others and change in clinical outcomes. Last, the variable instructor background and curriculum structure can be seen as an impediment to generalizability. However, the pragmatic approach of allowing each site to adapt the curriculum based on their resources will make it easier for other residency programs to adopt the EPEC-N curriculum.

### Lessons Learned

#### Neuropalliative Care Can Be Taught Without Neuropalliative Care Specialists

Despite none of our instructors having had fellowship training in palliative medicine, they were able to adapt and implement a neuropalliative care curriculum when given expert-written materials. Learners had a positive response and felt that their knowledge had improved, and instructors were enthusiastic about continuing to use the curriculum materials.

#### Understanding Learners' Needs and Preferences Is Key

In open-ended comments, several learners highlighted their preference for case-based and interactive learning that focuses on practical concepts and skills for patient care. This is consistent with a national survey of neurology residents showing that residents desire both a greater quantity and quality of palliative care skills training, particularly active and experiential learning methods.^21^ Although the EPEC-N curricular materials focus on interactive lectures, there is an opportunity to modify the materials to include more case-based discussion, role-play, and even simulated patient encounters. Our results suggest that teaching in small groups of learners may also have a positive effect in teaching efficacy. Last, although 2 of 3 sites used some type of precurricular needs assessment to select module topics, a more expanded needs assessment that accounts for other sources of palliative care education may be helpful.

#### Faculty Development Is Needed Around Both Neuropalliative Care Content and Teaching Techniques

Although our instructors did not have formal postgraduate training in palliative care, most had previous clinical experience (such as a clinical rotation in palliative care) or other training experience, such as the Become an EPEC Trainer conference, which covers both general palliative care topics and teaching techniques rooted in adult learning principles.^19^ Learners had a mixed opinion on whether the teaching formats were effective, which further faculty development in teaching techniques may address. To replicate the successful dissemination of the original EPEC curriculum, a train-the-trainer workshop specific to EPEC-N was piloted in 2022 and reached 26 participants from 19 institutions. Successful dissemination and uptake of EPEC-N will likely require ongoing expansion of this program. As more neurologists (and members of the interdisciplinary team) are called on to teach neuropalliative care, we would encourage them to pursue further training in both palliative care topics and specific teaching techniques. A variety of faculty development options are available, including through EPEC and VitalTalk.

Overall, the EPEC-N modules are a resource that can be easily adapted for use in neurology residency programs, mitigating multiple barriers to neuropalliative care training, including availability of expert faculty and time needed to develop teaching materials. There are many areas for potentially fruitful future work. Further study is needed to define the optimal methods and strategies for delivering neuropalliative care skills education. Possible improvements might include integration with other existing curricula such as VitalTalk, which may offer both palliative care skills training and practical knowledge.^22,23^ The EPEC-N modules and any other neuropalliative care education resource should be expanded to include topics on cultural and linguistic considerations for communication. Finally, to optimize patient care, neuropalliative care education must reach currently practicing neurologists and palliative care specialists, as well as nurses, pharmacists, advanced practice providers, and the entire interdisciplinary care team.

## Appendix Authors

**Table:**

Name	Location	Contribution
Eileen Harrigan, MD	Division of Geriatric Medicine and Palliative Care, Department of Medicine, NYU Langone Health, New York	Drafting/revision of the manuscript for content, including medical writing for content; major role in the acquisition of data; study concept or design; analysis or interpretation of data
Breana L. Taylor, MD	Department of Neurology, University of Washington, Seattle	Drafting/revision of the manuscript for content, including medical writing for content; major role in the acquisition of data; study concept or design; analysis or interpretation of data
Hannah L. Kirsch, MD	Department of Neurology & Neurological Sciences, Stanford University School of Medicine, Palo Alto, CA	Drafting/revision of the manuscript for content, including medical writing for content; major role in the acquisition of data; study concept or design; analysis or interpretation of data
Shivani Ghoshal, MD	Department of Neurology, NewYork Presbyterian-Columbia University Irving Medical Center, NY	Drafting/revision of the manuscript for content, including medical writing for content; major role in the acquisition of data; analysis or interpretation of data
Kimberly T. Kwei	Department of Neurology, NewYork Presbyterian-Columbia University Irving Medical Center, NY	Drafting/revision of the manuscript for content, including medical writing for content; major role in the acquisition of data; analysis or interpretation of data
Kate T. Brizzi, MD	Division of Palliative Care, Departments of Neurology and Medicine, Massachusetts General Hospital, Boston	Drafting/revision of the manuscript for content, including medical writing for content; major role in the acquisition of data; study concept or design; analysis or interpretation of data
Claire J. Creutzfeldt, MD	Department of Neurology, and Cambia Palliative Care Center of Excellence, University of Washington, Seattle	Drafting/revision of the manuscript for content, including medical writing for content; major role in the acquisition of data; study concept or design; analysis or interpretation of data
Tarini Goyal, MD	Department of Neurology, New York Presbyterian-Columbia University Irving Medical Center, NY	Drafting/revision of the manuscript for content, including medical writing for content; major role in the acquisition of data; study concept or design; analysis or interpretation of data

## Glossary

EPEC-N: Education in Palliative and End-of-Life Care for Neurology
PGY: postgraduate year
SABI: severe acute brain injury
